# Electrochemical Evaluation and Phase-related Impedance Studies on Silicon–Few Layer Graphene (FLG) Composite Electrode Systems

**DOI:** 10.1038/s41598-018-19929-3

**Published:** 2018-01-23

**Authors:** Qianye Huang, Melanie J. Loveridge, Ronny Genieser, Michael J. Lain, Rohit Bhagat

**Affiliations:** 0000 0000 8809 1613 grid.7372.1 WMG, The University of Warwick, Coventry, CV4 7AL UK

## Abstract

Silicon-Few Layer Graphene (Si-FLG) composite electrodes are investigated using a scalable electrode manufacturing method. A comprehensive study on the electrochemical performance and the impedance response is measured using electrochemical impedance spectroscopy. The study demonstrates that the incorporation of few-layer graphene (FLG) results in significant improvement in terms of cyclability, electrode resistance and diffusion properties. Additionally, the diffusion impedance responses that occur during the phase changes in silicon is elucidated through Staircase Potentio Electrochemical Impedance Spectroscopy (SPEIS): a more comprehensive and straightforward approach than previous state-of-charge based diffusion studies.

## Introduction

Within recent years, the requirements for energy storage devices with higher capacity have become more prevalent in areas such as vehicle electrification. This has stimulated intense growth in lithium-ion battery (LIB) research on a global scale. Within a battery, the design and type of anode materials is one of the main factors that determine higher degrees of reversible storage capacity for lithium ions. Silicon (Si) remains a promising anode material for next generation LIBs, with a sustainable abundance and high gravimetric capacity (3579 mAh/g): almost 10 times that of the capacity of graphite^1^. This has made it the focus of considerable research efforts within the last decade. However, the well-documented problems, namely particle pulverisation caused by volume expansion and an unstable solid electrolyte interphase (SEI) layer, are yet to be successfully resolved for the commercialisation of Si-based anodes in batteries^2,3^.

Graphene has been shown to possess excellent electrical conductivity, a high surface area^4^, and is considered a promising enabler to enhance the electrochemical performance of Si electrodes^5^. Several studies have been conducted on hybrid silicon/graphene electrodes with long cycle life achieved^4,6–12^. However, most of these studies either focused on nano-sized Si particles or used complicated chemical methods (such as electrophoretic deposition and chemical vapour deposition) to combine silicon and graphene. Such approaches are considered impractical to progress to large-scale manufacture.

Additionally, due to its large surface area, the use of nano-sized Si would generate a higher capacity loss during the first charge cycle due to the formation of large amounts of SEI species on the particle surface^13^. This would deplete the Li inventory and subsequently reduce the number of possible charge-discharge cycles.

A previous study on these systems has reported that interconnecting few-layer graphene (FLG) with Si can be an effective solution to enhance the cycling stability through the formation of a conductive, hierarchical structure^14^. This approach was adopted in this study and all electrodes were manufactured with a relatively high mass loading of micron-sized silicon particles using mechanical dispersion apparatus aligned with industrial electrode fabrication techniques.

Electrochemical impedance spectroscopy (EIS) has been widely applied to analyse the underlying kinetics of electrodes, and the impedance of Si-based anodes^15–18^. However, most of the previous studies on Si-graphene systems were limited to one impedance measurement after a specified number of cycles^6,7,9^, which is inconclusive to confirm any improvements to resistance as provided by graphene, as the impedance value may vary against cycle number. In this study, Potentio Electrochemical impedance spectroscopy (PEIS) will determine the variation in impedance as a function of cycle number to demonstrate the influence of the FLG on the resistance magnitude. A number of state-of-charge (SoC) based diffusion studies have also been conducted on Si, but they neither found the connection between impedance behaviour and lithiation level of Li_x_Si electrodes^19^, nor compared the diffusion variation as a function of cycle number^20,21^. Staircase PEIS (SPEIS) is a useful technique to measure the impedance at different voltage steps within a lithiation/delithiation cycle. For each measurement, it is easy to distinguish the diffusion related Warburg impedance by splitting the frequency range. This with existing knowledge of Si phase changes at corresponding voltage steps makes the direct relation between Si phase change and diffusion impedance more obvious. This will also allow a better understanding of the diffusion parameters critical to the cycling performance.

## Result and Discussion

### Scanning Electron Microscope (SEM) imaging

**Figure 1:**
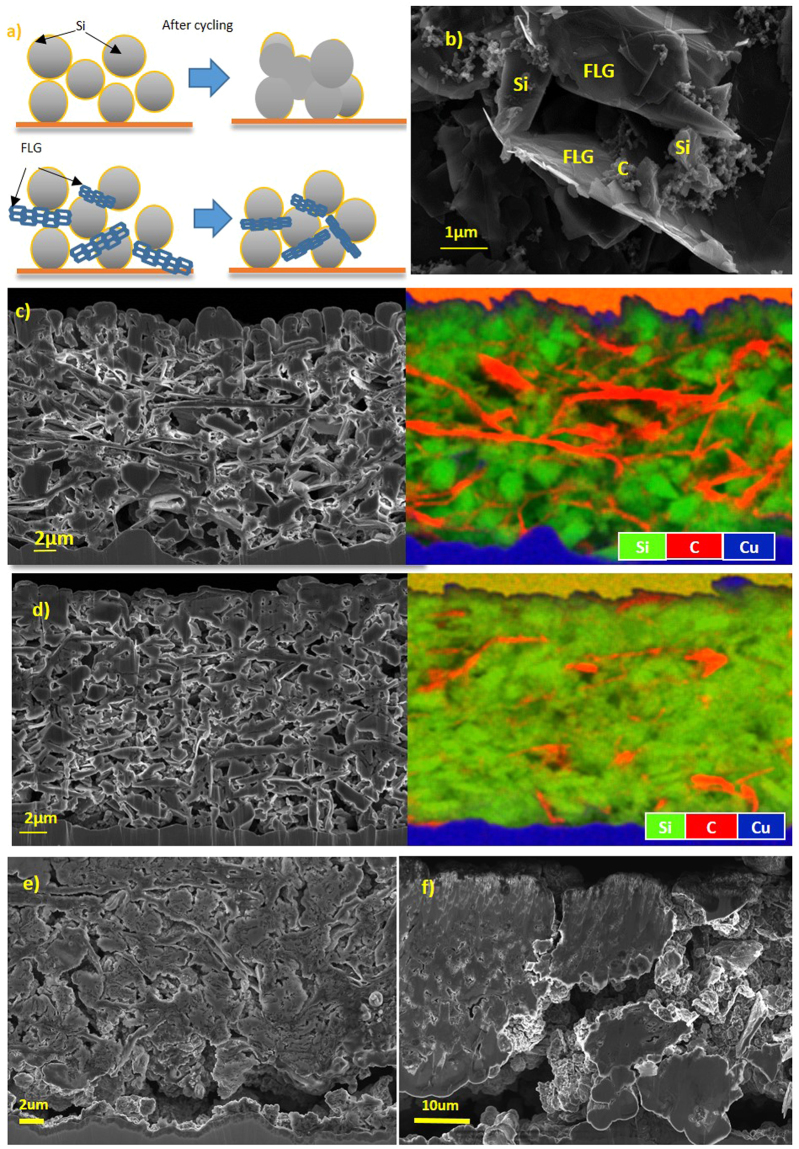
(**a**) Schematic of FLG preventing Si electrochemically “fused” together; (**b**) SEM image for Si-FLG electrode (60% Si: 16%FLG: 14% Na-PAA: 10% Carbon mix); (**c**) Cross-section image and EDS mapping for Si-FLG composite electrode; (**d**) Cross-section image and EDS mapping for Si only electrode; (**e**) Cross-section image for Si-FLG electrode after 100 cycles and (**f**) Cross-section image for Si only electrode after 100 cycles.

Figure 1 shows the microstructure change for Si and Si-FLG composite electrode (Formula D, as specified in Table 1). Figure 1a shows the mechanism of how FLG helps to mitigate the Si particles electrochemically alloying together through electrochemical fusion. Figure 1b shows that the particles in the Si-FLG electrodes (Formula D, as specified in Table 1) are evenly distributed, and have formed a hierarchical network between the Si, FLG and carbon black. Within this network, we suggest that FLG augments long-range planar conductivity while carbon black provides short-range conductive pathways between the graphene layers and Si particles.

**Table 1:** Si/graphene formulation matrix.

**Formulation**	**Mass ratio %**			
	**Si**	**Few-layered Graphene**	**Na-PAA**	**Carbon mix**
**A**	76	0	12	12
**B**	70	8	12	10
**C**	65	12	14	9
**D**	60	16	14	10

Cross-section SEM images and energy dispersive X-ray spectroscopy (EDS) mapping (Fig. 1c,d) show that by incorporating FLG into the Si-based anode, the density becomes ~0.42 g/cm^3^, which indicates a higher porosity in comparison with the Si-only electrodes, which have a density of ~0.75 g/cm^3^. It can be observed from Fig. 1e,f that, in the Si-only electrode, Si particles are mostly agglomerated within larger Si masses with non-distinct boundaries. While for Si-FLG electrodes, Si particles remain clearly separated by FLG sheets with particle size ranges consistent with the volume variations. The implication of this is that when Si particles become merged in this way then Li diffusion pathways are effectively increased in such regions and particles deep within the fused regions may contain electrically isolated Li^22^.

### Cyclic electrochemical performance

**Figure 2:**
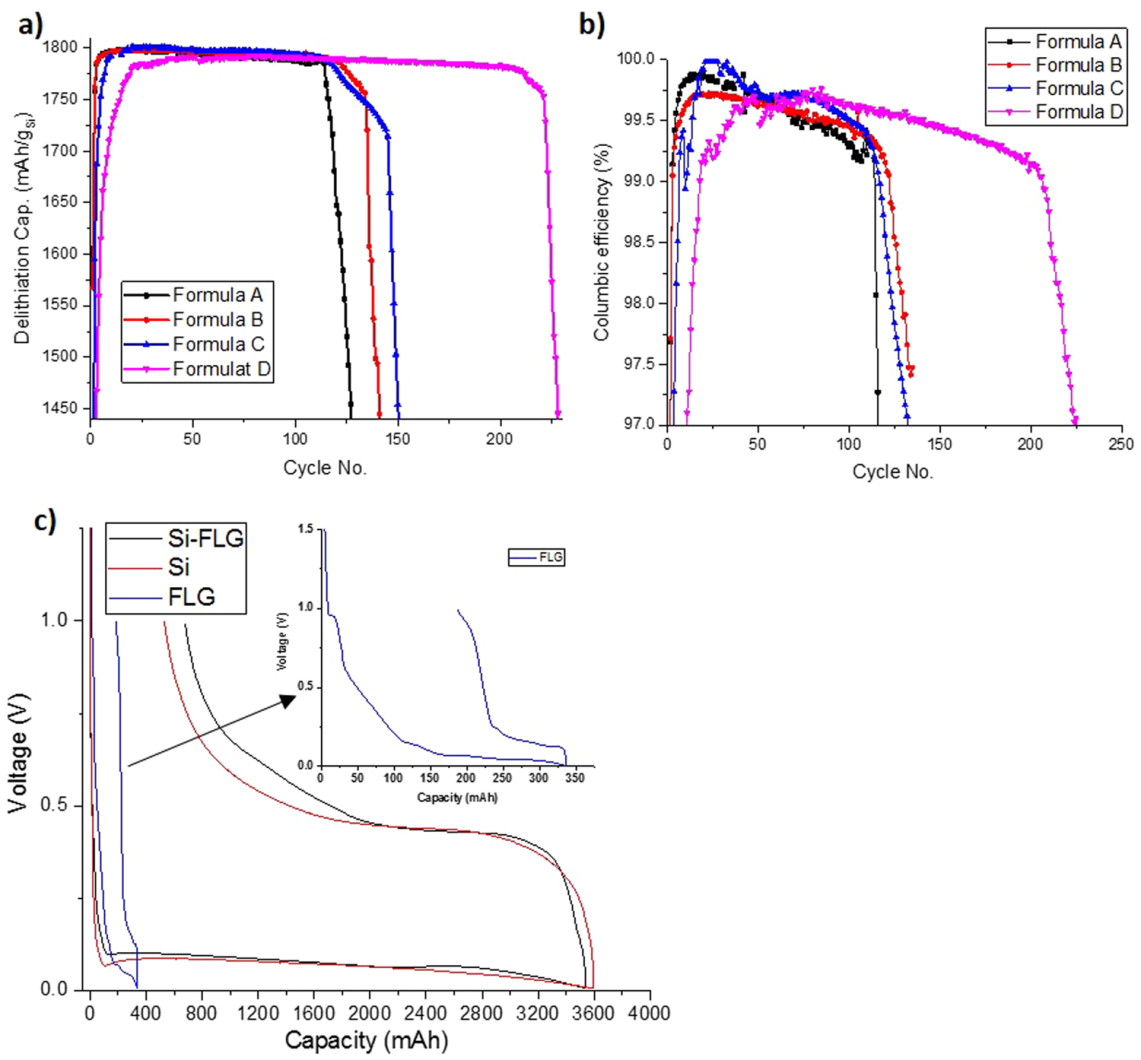
(**a**) Half-cell cyclability and (**b**) columbic delithiation efficiency under capacity limitation of 1800 mAh/g for Si-FLG formulation matrix (Formula A – 76% Si: 0% FLG: 12% Na-PAA: 12% Carbon. Formula B – 70% Si: 8% FLG: 12% Na-PAA: 10% Carbon. Formula C – 65% Si: 12% FLG: 14% Na-PAA: 9% Carbon. Formula D – 60% Si: 16% FLG: 14% Na-PAA: 10% Carbon). (**c**) First cycle voltage profile for half-cells of Si-FLG with Formula D, Si only and FLG only electrodes cycled between 1 V-5 mV without capacity limitation.

The half-cell (vs. Li foil) cyclability of the Si-FLG electrodes using different formulations, and the comparison of the first cycle voltage profile for electrodes based only on Si only, Si-FLG and FLG only are shown in Fig. 2. It can be observed from Fig. 2a that initially all half-cells are cycled stably at 1800 mAh/g specific lithiation capacity (based on the active mass of Si), but they fade dramatically at a certain point of cycling, which is normally referred to as the “roll-over” effect^23^. This can be caused by either electrode deterioration or the continued growth of SEI layer on the surface of Si becoming thick enough to resist Li-ion penetration^24^. Figure 2b shows that the cyclic coulombic efficiency profile, which increases after formation of the initial SEI layer, and subsequently decreases due to the side reactions with electrolyte, continuously forming products such as lithium fluoride and lithium carbonate^25^. Amongst the matrix Formulation D, which contains the highest proportion of FLG (60% Si: 16% FLG: 14% sodium-polyacrylic acid (Na-PAA): 10% carbon mix), delivered the best performance in terms of cycle life and coulombic efficiency, with 80% capacity retention after 230 cycles. It indicates that by incorporating FLG, the “roll-over” effect is significantly delayed, possibly due to the enhanced tensile properties from the FLG (Supplementary Figure 1), which reduces the rate of the pulverization of the electrode microstructure. When referring to the previous Si-FLG study^14^, which demonstrated about 200 cycles under the capacity 1800 mAh/g, it can be concluded that 16% FLG in this formulation is a more effective ratio.

In order to investigate the capacity that FLG can contribute within this voltage range, Si-FLG (Formula D) was compared with Si only (76% active mass) and FLG only (76% active mass) under the same current density and without capacity limitation. Figure 2c indicates that with the same current density of 90 mA/g and 5 mV cut-off voltage, the FLG only electrodes (76% active mass) deliver a maximum contribution of around 330 mAh/g, with a large initial capacity loss of about 180 mAh/g. The capacity achieved by the FLG-only electrodes is much lower than the result in a previous study^14^ and this could be due to the current density (90 mA/g) being significantly increased during the first cycle. The large initial capacity loss is attributable to the surface area of FLG, resulting in large quantities of lithium ions becoming irreversibly consumed in the SEI layer.

### Differential capacity analysis

**Figure 3:**
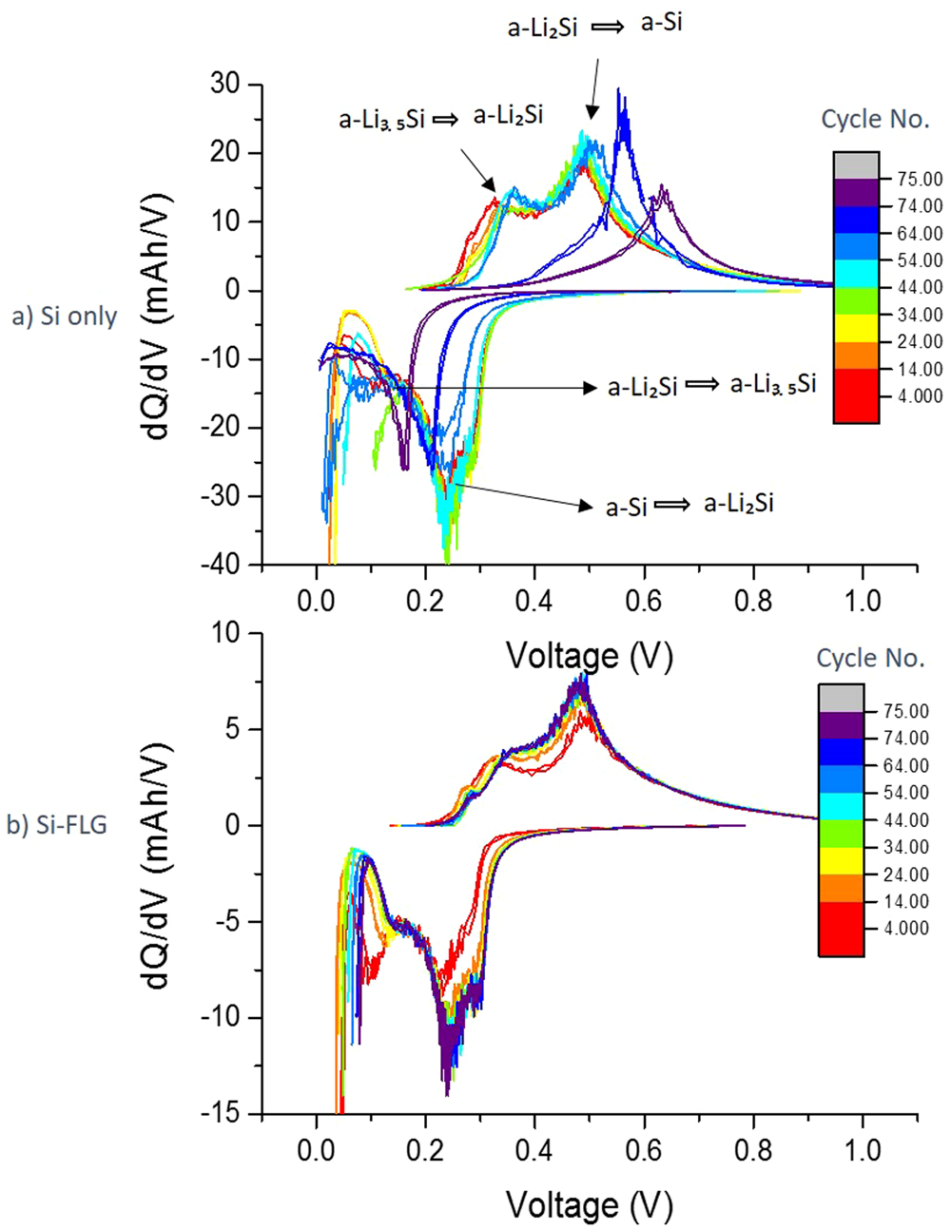
dQ/dV plot for cycle 2–60 of **(a)** Si only electrodes **(b)** Si-FLG electrodes.

The differential capacity (dQ/dV) plots of Si only and Si-FLG half-cells for different cycle numbers are shown in Fig. 3. The peaks during the (de)lithiation process correspond to different phase equilibria of Li_x_Si. It can be observed from Fig. 3a that Si-only and Si-FLG electrodes have similar dQ/dV quasi-plateaus, outlined in Supplementary Table 4^10,14,26^, but the plateaus for Si-FLG electrodes are broader. This relates to the quasi-plateaus for Si and FLG components within the electrode overlapping with each other. It can further be seen that for the Si only electrodes, there are obvious reductions in lithiation plateaus and voltage shift around 0.25 V during lithiation and around 0.5 V during delithiation after 50 cycles. It is observed that at this point plateaus become single plateaus for both lithiation and delithiation process. This is possibly related to the resistance on the surface of the electrodes is increasing due to the thicker SEI layer, resulting in an increase in overpotential, which delays the lithiation process. In this case, with the 5 mV cut-off voltage limitation, Si electrodes cannot be further lithiated to a-Li_3.5_Si^27^. For the Si-FLG electrodes, there are no obvious plateau reductions or peak shifting. This supports the observation that Si-FLG composite electrodes show longer cycle life and reduced voltage hysteresis due to a lower resistance, compared with electrodes based only on Si as the active material.

### Potentio Electrochemical Impedance (PEIS) study

**Figure 4:**
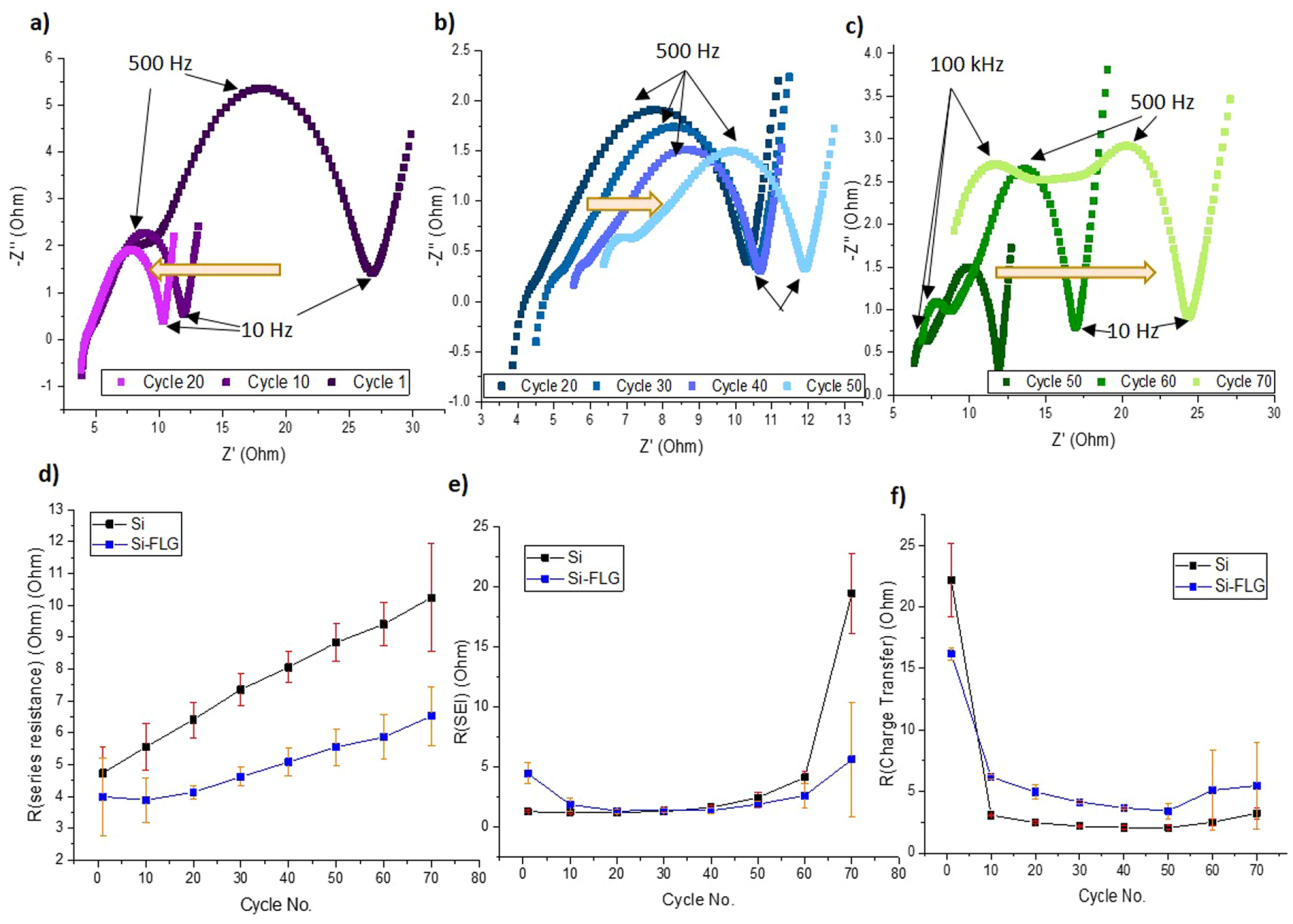
Nyquist plots of Si-FLG half cells at 50% SoC during charge process from **(a)** cycle 1 to cycle 20; **(b)** cycle 20 to cycle 50; **(c)** cycle 50 to cycle 70; impedance fitting result comparison of **(d)** series resistance **(e)** SEI resistance **(f)** interphase contact and charge transfer resistance.

Figure 4a–c shows the impedance spectra for the Si-FLG half-cells for different cycle numbers, and Fig. 4d–f compares the fitting results over numbers of cycles between Si only and Si-FLG half-cells based on the equivalent circuit (Supplementary Figure 2). The impedance values lie in a reasonable range for coin cells, as compared with a commercial graphite electrode (LiFun Technology, China) (Supplementary Figure 3). It can be seen that for Si-FLG half-cells from the 1^st^ to 20^th^ cycle, there is a small semicircle related to SEI-derived resistance, but a clear decrease for the second semi-circle that relates to charge transfer resistance. This is in agreement with previous studies^16^ and has been explained by the volume expansion of Si, causing the electrode to be pressurised by the spring inside the coin cell. This results in a better electrical contact between the active material/binder system and current collector, leading a decrease of interphase electronic contact resistance. From the 20^th^ to 50^th^ cycle, it is observed that the series resistance starts to increase, indicating that the ionic conductivity of the electrolyte or electrical conductivity through electrodes or current collector becomes reduced. Additionally, the SEI-related semicircle increases gradually, which is possibly caused by the blocking of electrolyte channels, arising by the way of microstructural changes in the electrode. It is feasible that the electrochemical fusion of Si particles or decomposition of the electrolyte are likely phenomena here^22^. The interphase contact and charge transfer resistance remains stable but after the 50^th^ cycle, the SEI resistance grows dramatically, which indicates an inhibited transport of lithium ions through this layer.

Comparing the composite Si-FLG with the Si-only electrodes, Fig. 4d shows that generally the Si electrodes show a higher series resistance than Si-FLG, which grows more dramatically during subsequent cycling. This is because without the FLG, Si particles are more likely to grow into an electrochemically welded state during cycling, as outlined previously. Thus it becomes more difficult for Li ions to access the surface of Si particles^22^. As the FLG facilitates and maintains Si particle separation in electrodes, the electrolyte channels remain unobstructed. Additionally, since the series resistance is defined by the internal electronic resistance of the electrode, a lower series resistance here indicates enhanced electronic pathways inside the electrode. From Fig. 4e initially, the Si-FLG electrodes display a larger SEI resistance than the Si electrodes due to a larger surface area. After 50 cycles, the SEI resistance increases significantly for both electrodes, caused by continual electrolyte decomposition. The relatively slower growth of SEI resistance in Si-FLG may simply reflect on there being proportionally less Si in Si-FLG electrodes. The SEI layer of FLG is quite stable during (de)lithiation, which improves the general performance stability of the electrode. Figure 4f shows the trend for interphase electronic contact and charge transfer for both electrodes (with similar performance), as explained previously.

### Staircase Potentio Electrochemical Impedance Study (SPEIS)

**Figure 5:**
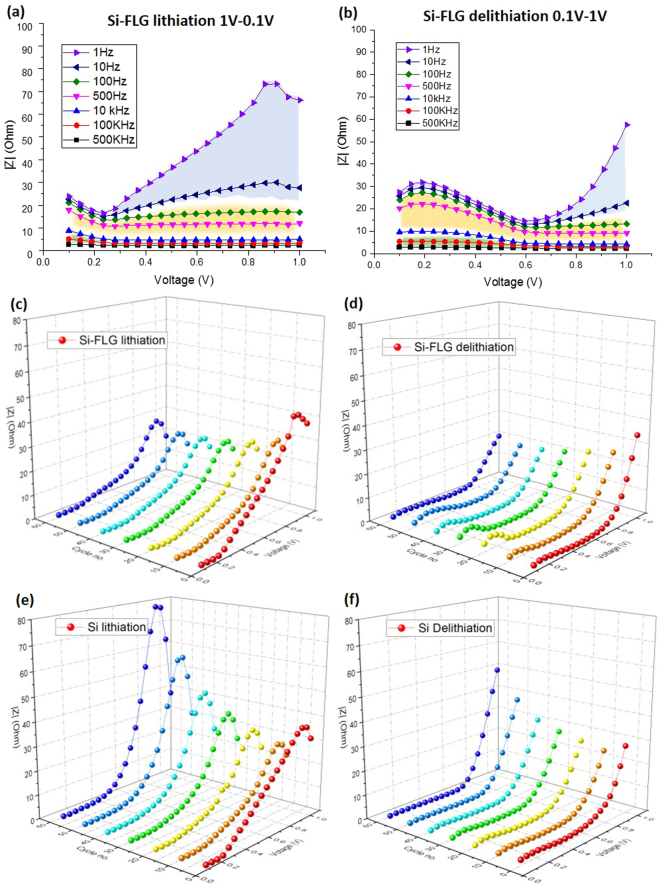
SPEIS result of Si-FLG after the first cycle for (**a**) lithiation process and (**b**) delithiation process; diffusion impedance at 1 Hz for Si-FLG (**c**) during lithiation and (**d**) delithiation process; and for Si (**e**) during lithiation and (**f**) delithiation process.

In order to further investigate the diffusion resistance, SPEIS study has been conducted, which focuses on the impedance variation as function of a voltage and, by splitting the frequency range, makes it more straightforward to investigate the low frequency impedance (including the diffusion related Warburg impedance). Figure 5a,b show the SPEIS result for the Si-FLG electrodes (Formula D) following the formation cycle for lithiation (Fig. 5a) and for delithiation (Fig. 5b). In each graph, there are selected frequency ranges from 1 Hz to 500 kHz. As marked in Fig. 4a–c, high frequency impedance responses (at 10 kHz to 500 kHz) are attributable to the SEI resistance and the series resistance (highlighted green areas in Fig. 5b). Impedance responses between 10 Hz and 10 kHz (orange area) represent the interphase electronic contact and the charge transfer resistance. Impedance responses with frequencies less than 10 Hz (blue area) are termed as Warburg impedance, which is mainly determined by diffusion coefficient, is also termed as diffusion impedance in this study^28^.

Figure 5a shows that during the lithiation process, the impedance values under the high AC frequency range (series and SEI resistance) remain stable throughout the voltage range, which is in agreement with previous findings^29,30^. This is because the conductivity of the electrolyte does not change significantly and there is no pronounced decomposition of the electrolyte. Whilst the interphase contact and charge transfer resistance is reasonably stable - starting from 1 V and reaching 0.25 V - it increases at voltages below 0.25 V. This relates to the Si phase change from a-Si to a-Li_2_Si and affects the exchange current density, which is a materials-dependent parameter that determines the charge transfer resistance. For the Warburg impedance, there is an initial increase followed by a sizeable decline becoming stable at around 0.25 V. The initial increase is possibly because after the first cycle, the relaxation time (10 minutes) is insufficient to ensure that all the Li^+^ has left the Si electrode. The remaining Li^+^ provides additional resistance for the subsequent lithiation process. The diminished diffusion resistance may be explained by the Si phase change during the (de)lithiation process. Initially, Si particles mostly exist as “cage-like” crystalline structures, which largely constrain the movement of Li atoms. As the c-Si is lithiated to a-Li_2_Si, more and more Si-Si bonds are broken, which release the constrained Li atoms, thus reducing the resistance of Li diffusion. This process has been investigated and clarified by Key *et al*. in an NMR study^10^ and other SoC-based diffusion studies on Si, concluding similar results^21^. Additional evidence is the “stopping point” for diffusion impedance decreases, occurring at around 0.25 V, and coincident with the first lithiation plateau in the dQ/dV plot, representing the phase change equilibria.

For the delithiation process, the trends in the impedance data are similar in reverse to those in the lithiation process, and the “starting point” of the impedance increase (around 0.6 V) relating to the plateau of delithiation in the dQ/dV plot. This relates to the growth of Si clusters. Additionally, the diffusion impedance after 0.6 V is lower during the delithiation process as compared with lithiation. This is because following the Si lithiation to an amorphous phase from crystalline during the first cycle, it will be only delithiated to an amorphous phase in the subsequent cycles (never reverting back to crystalline^10^). These results indicate that the impedance variation is highly phase-change correlated.

Additionally, it can be concluded from Fig. 5a,b that it is preferable to take the cyclic PEIS measurement above 0.25 V during the lithiation process, because the impedance value under medium and high frequencies is quite stable with a large enough window to account for the voltage shift during cycling. We mainly use PEIS studies to compare the cyclic series resistance and charge transfer resistance to better understand the degradation mechanism - which occurs in the medium and high range frequency. A stable impedance value within this frequency range is thus essential (despite voltage variability) but previous impedance studies on Si have rarely addressed this issue.

Figure 5c–f compares the SPEIS result under 1 Hz (the blue area which represents the diffusion impedance), from Cycle 1 to Cycle 60 for both the Si-FLG and Si only electrodes. It is clear that both electrodes have a similar impedance variation trend against the voltage, but the peak diffusion impedance for Si-FLG electrodes is smaller than that of Si-only electrodes after 20 cycles. Due to the poorer cycling performance and more significant voltage hysteresis for the Si only electrodes, it is more likely to drop further below 50 mV to reach a capacity of 1800 mAh/g. In this case, more metastable c-Li_15_Si_4_ will be formed in the Si-only electrodes (compared with Si-FLG), which is more resistant to delithiation back to a-Li_1_._1_Si due to more tough Si-Si bonds^10^. As the pulverization of the electrode progresses, it is possible that some Si clusters become isolated from subsequent lithiation processes, thus further contributing to capacity loss.

From cycle 1 to 10, the peak diffusion resistance for both electrodes becomes largely decreased. According to the voltage profile in Supplementary Figure 4, the lithiation in the first cycle has been cut off at around 0.1 V, by which point there could be some crystalline Si remaining within the electrode. Therefore, for the first SPEIS measurement after cycle 1, the remaining crystalline Si clusters are involved in the lithiation process, which shows relatively high diffusion impedance. After an additional 10 constant current (CC) cycles, for the second SPEIS measurement, the Si clusters are mostly lithiated to amorphous phases, which are less resistant to diffusion. This can explain why there is more diffusion resistance in the 1^st^ cycle than in subsequent cycles.

## Conclusion

This study comprehensively investigated the performance of Si-FLG composite electrodes, manufactured by an industrially relevant methodology. Electrochemical evaluation and phase-related impedance spectroscopy were the main investigative tools. The reversible electrochemical performance has demonstrated that by incorporating FLG into a high mass % content of Si, the cyclability is largely improved. In addition, it was demonstrated that FLG could contribute a capacity of around 300 mAh/g under the high limited capacity condition (1800 mAh/g). We recommend that the ratio of FLG to Si needs to be limited due to the significantly large first cycle loss relating to the large surface area of the FLG.

With PEIS and SPEIS, it was shown that FLG helps Si to maintain an overall lower series resistance during cycling. It could also confer performance benefits through some prevention of the Si particles from agglomerating though electrochemical fusion – but this phenomenon warrants further, systematic investigation. Additionally, the improved cyclability of Si-FLG keeps the internal diffusion impedance at a relatively stable level. Combining the dQ/dV analysis with SPEIS, this study has addressed that the diffusion impedance in Si is highly phase dependant. On the other hand, it indicates that SPEIS could be a more straightforward method to study the change of diffusion impedance with different voltage as well as to investigate the diffusion impedance as a function of cycle number. Finally, the SPEIS results suggests that for the Si-based electrodes, a voltage range with a stable impedance is essential and so it is more reliable to take the PEIS measurement above 0.25 V during the charge process.

## Methods

### Electrode materials

Silicon powder (d_50_ = 3.1 µm, purity 99.7%) was obtained from Elkem (Silgrain e-Si). Few-layer graphene (FLG) was obtained from XG Sciences (xGnP-M5, 6–8 nm thickness). Raman analysis in our previous study has concluded that the number of layer was greater than or equal to five^14^. The composite carbon blend includes carbon black, graphite and few-layer graphene to provide a hierarchical conductive network (Supplementary Table 1). The binder used in this study is partially neutralised Polyacrylic Acid (PAA) solution (See supplementary), which provides effective hydrogen bonding between particles^31^.

### Electrode manufacture

To investigate the effect of the FLG to the cyclability of Si anodes, a matrix formulation study was conducted with four different formulations to vary the ratio between the Si and FLG (Table 1).

The electrode materials included active material powder, a mixture of carbons and deionised water, with mass proportions according to their designated formulation. The slurry was initially mixed using an overhead high-speed homo-disperser (Model 2.5, PRIMIX) at 1000 rpm for 30 minutes. Following this the slurry was ultrasonically processed (UP400S, SciMED) for 7 minutes at 60% amplitude, followed by manual stirring with a spatula and subsequent ultra-sonication for a further 7 minutes. The binder was added into the slurry and mixed using a high-speed homo-disperser at 1000 rpm for 30 minutes. Finally, the mixed slurry was transferred to a Filmix (high shear processor, Model 40–40, PRIMIX) to ensure a homogeneous distribution of the nano-sized particles and to prevent secondary agglomeration. The solution was processed at the speed of 10 m/s for 30 s, then 25 m/s for another 30 s.

The resulting slurry was coated onto copper foil (10 μm, Oak Mitsui, electrodeposited) using a draw-down coater (RK Instruments Ltd) with a micro-meter-controlled spreading blade (K control coater Model 101, RK Print) with a blade gap of 80 μm. The coated copper foil was dried on a hot plate set to 50 °C to evaporate the solvent for 10 minutes. The coated sheet was placed in a vacuum oven set to 50 °C for *ca*. 12 hours to ensure minimizing of any water prior to cell construction.

To investigate the electrochemical performance of Si-FLG electrodes, electrodes based only on either Si or FLG were manufactured based on formulations in Supplementary Table 2.

### Electrochemical characterisation

The electrodes were assembled into Hohsen CR2032 coin cells in a dry room with dew point of −45 °C to reduce water content from the electrode. Lithium foil (D = 15.6 mm, PI-KEM) was used as counter electrode and a trilayer membrane (PP-PE-PP, Celgard) was used as separator. The cells were filled with a commercially available electrolyte, which was 1 M LiPF_6_ dissolved in solvent of EC/EMC (1/3), 15% FEC, and 8% VC (PuriEL, Soulbrain).

The half-cells were cycled at constant current mode between 5 mV and 1 V on Maccor Series 4000 system with a fixed C-rate at C/20 (90 mA/g) for the formation cycle, followed by repeated cycling at the C-rate of C/5 (360 mA/g). For Si-FLG formulation matrix study and comparison differential capacity analysis, cells are also cycled under a capacity limitation of 1800 mAh/g based on around 2 mg active mass.

### Electrochemical Impedance Spectroscopy

To demonstrate improvements in conductivity by the incorporation of FLG, SPEIS test and PEIS test were carried out using a VMP3 potentiostat (Bio-Logic).

The PEIS test was used to measure the impedance change as a function of cycle number. The test was conducted with voltage amplitude of 10 mV, measured between frequencies of 500 kHz and 100 mHz at 50% SoC of each cell. The first measurement was taken after the formation cycle with additional 10 minutes relaxation time, and repeated with every 10 cycles. The impedance fitting equivalent circuit and explanation for relevant elements are shown in Supplementary Figure 2.

The SPEIS test in this study measured impedance at different voltage steps within one cycle. Half-cells with Si-FLG, Si only and FLG only electrodes were charged and discharged using the same constant current mode and measured for every 10 cycles. SPEIS was conducted with a staircase potential swept from 1 V to 0.1 V with 20 steps (10 minutes rest after reaching each voltage step) during lithiation, followed by another SPEIS measurement repeated from 0.1 V to 1 V during delithiation. The voltage profile is shown in Supplementary Figure 4.

## Electronic supplementary material

Supplementary information
