# Supplementary material for: Electrochemical Evaluation and Phase-related Impedance Studies on Silicon–Few Layer Graphene (FLG) Composite Electrode Systems
Source: Sci Rep. 2018 Jan 23;8:1386. doi: 10.1038/s41598-018-19929-3 (PMC5780504; doi:10.1038/s41598-018-19929-3)
Supplement: Supplementary file 1 — Supplementary information [file 41598_2018_19929_MOESM1_ESM.pdf]

Electrochemical Evaluation and Phase-related Impedance Studies on Silicon–Few Layer Graphene (FLG) Composite Electrode Systems

Qianye Huang*, Melanie J. Loveridge, Ronny Genieser, Michael J. Lain and Rohit Bhagat

Supplementary method and figures

Multi-Carbon Solution

Carbon black (SUPER C65, C-NERGY), graphite (SFG6, TIMREX), and Few-layer Graphene (FLG) were weighted according to the formulation listed in Table 1. The mixture was initially hand-blended by for 5 minutes, prior to medium-sheer processing using an overhead high-speed homo-disperser (Model 2.5, PRIMIX) at 1000rpm for 30 minutes. The resulting slurry was further processed using an ultrasonic probe (UP400S, SciMED) for 15 minutes using an amplitude of 60% and a frequency of 24Hz, followed by further dispersion using a high-speed homo-disperser for 30 minutes at 1000rpm.

Table 1 Carbon mix formulation

Carbon mix	Mass (g)	wt.% Solids
**Carbon Black**	5	2.6
**FLG**	10	5.2
**Graphite**	5	2.6
**Sodium-Polyacrylic Acid (Na-PAA, 12.5% concentration)**	1	0.5
**Deionised water**	169.91	
**Total Mass**	190.91	10.9

Binder Solution

The Polyacrylic Acid (PAA) powder (MWT = 450 K, purity ≥ 99.5%) was purchased from Sigma Aldrich. 13g PAA powder was dissolved in 87g of deionised water using a Turbula (T10B Glen Mill) for 24 hours, followed by an overnight degassing period.

For the partial neutralisation, 5g sodium hydroxide pellets (NaOH, Sigma-Aldrich) were dissolved in deionised water added to the above PAA solution and blended using a spatula for 10 minutes. The resulting partially neutralised PAA solution was allowed to de-gas for 24 hours before it could be used.

Electrode Comparison Study

For further comparison with the forth formulation, bare Si and bare FLG electrodes were made according to the formulation in Table 2.

Table 2 Formulation for further comparison study

Electrode	Mass ratio %			
	Si	Few-layered Graphene	Na-PAA	Carbon mix
**Si/FLG hybrid**	60	16	14	10
**Si only**	76	-	14	10
**FLG only**	-	76	14	10

SEM and Cross Section Image

Carl Zeiss Sigma Field Emission Scanning Electron Microscope (FE-SEM) was used to take the SEM image of electrodes and cross section under the voltage of 5kV and an in-lens detector. The working distance was about 2.5mm.

Cross-sections were obtained through HITACHI Ion Milling System (IM4000 plus) at an acceleration voltage of 6 kV for 10 minutes.

The cycled cells are dissemble in the dry room and washed with dimethyl carbonate (DMC), and then follow the same procedure as stated above to obtain the cross-section images.

Electrode Density

The electrode density was obtained through measuring the mass and the thickness of the electrode. The area of electrode is known as 1.72cm^2, so the electrode density ρ can be calculated by:

$$\rho = \frac{mass}{1.72 \times thickness}$$

Nano Indentation Test

The nano-indention test is conducted on nano-indenter (NanoTest Extreme, Micro Materials Ltd, UK) with a Berkovich indenter which diameter is 25um.

The test process is:

1. Applied the load to the electrode until it reached 15mN.
2. Hold it for 300s to ensure the creep exponent has been removed during unloading.
3. Remove the load and thermal drift correction for 60s.

The tensile curves are shown as **Figure 1**. Hardness and modulus are calculated using data taken from the slope of the tangent to the unloading curve, summarized as **Table 3**. It can be observed

from Table 3 that the Young's modulus has been significantly reduced with incorporating FLG into Si electrode.

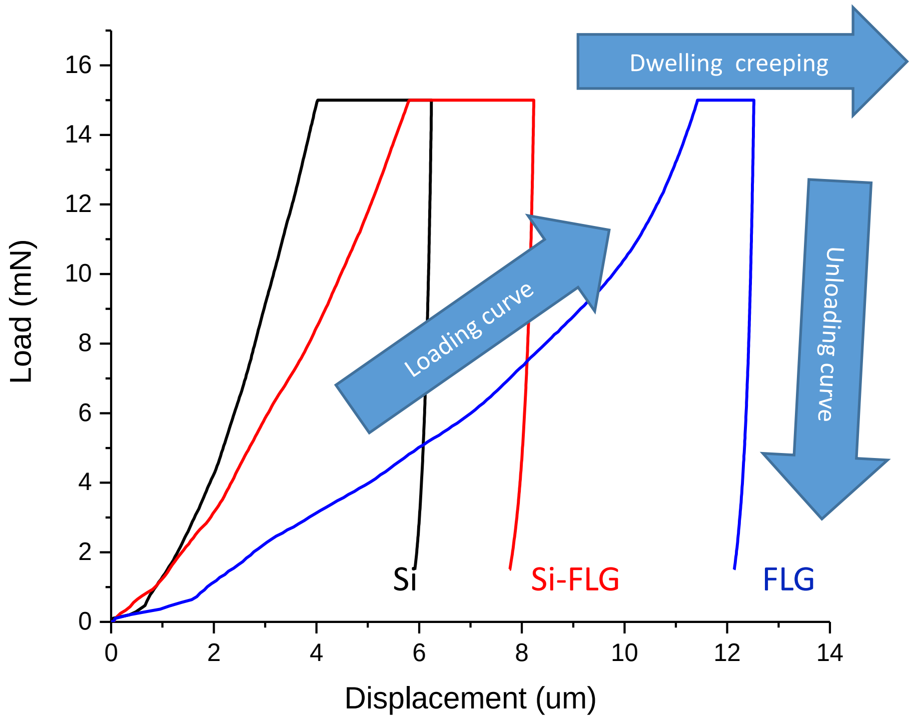

Figure 1 Tensile curves from nano-indentation test

Table 3 Maximum contact depth, hardness and Young's modulus achieved from nano-indentation test

Sample	Maximum Depth (um)	Hardness (GPa)	Reduced Young's Modulus (GPa)
Si	6.22±0.33	0.016±0.002	2.38±0.19
Si-FLG	7.75±0.84	0.010±0.002	1.42 ±0.27
FLG	12.37 ± 0.93	0.004±0.001	1.06±0.11

Phase Change Process for dQ/dV quasi-plateaus

Table 4 *Corresponding phase change at voltage peaks*[1–3]

Process	Peak voltage	Phase change (after 2nd cycle)
**Lithiation**	0.25 V	a-Si → a-$Li_{2.0}Si$
	0.1 V	a-$Li_{2.0}Si$ → a-$Li_{3.5}Si$
	≤50mV	a-$Li_{3.5}Si$ → c-$Li_{15}Si_4$

**Delithiation**	0.25 V	a-$Li_{3.5}Si$ → a-$Li_{2.0}Si$
	0.5 V	a-$Li_{2.0}Si$ → a-Si
	0.45V	c-$Li_{15}Si_4$ → a-$Li_{1.1}Si$

PEIS Result Fitting Typically, the Nyquist plot of electrodes in a Li-ion battery contains two semi-circle and a linear drift of 45˚ to follow. It has been widely agreed that the intersection point of the X-axis refers to the series resistance, which is also called electrolyte resistance[4,5]. The first semi-circle represents the resistance when Li-ions are diffusing through the SEI layer, and the second refers to the charge transfer at the interface between the electrolyte and the active material. However, previous impedance studies on Si based electrodes[6, 7] have suggested that interphase electronic contact resistance between the active material and the current collector (which normally reacted at medium-high frequency range) should be also taken into consideration. The straight-line tail followed is referred to as Warburg impedance, which includes the diffusion impedance of Li ions within active material. Based on the EIS spectra of the electrodes in this study and the previous literatures, the fitting equivalent circuit is shown in **Figure 2**. It consists of a resistor representing series resistance, and is followed by a series of three resistors in parallel with a constant phase elements and a Warburg diffusion element at the end. They respectively account for the SEI resistance, interphase electronic contact resistance and charge transfer resistance.

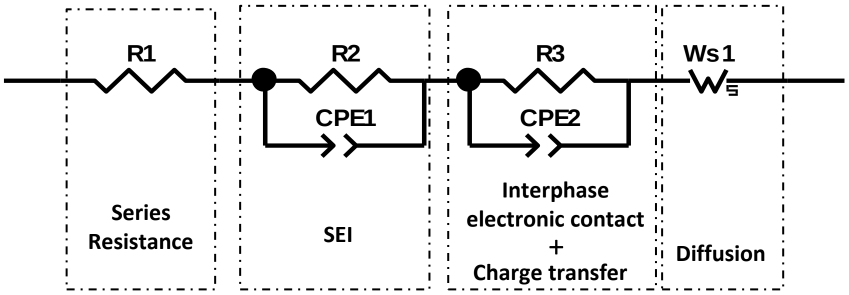

Figure 2 The equivalent circuit for fitting the impedance spectra

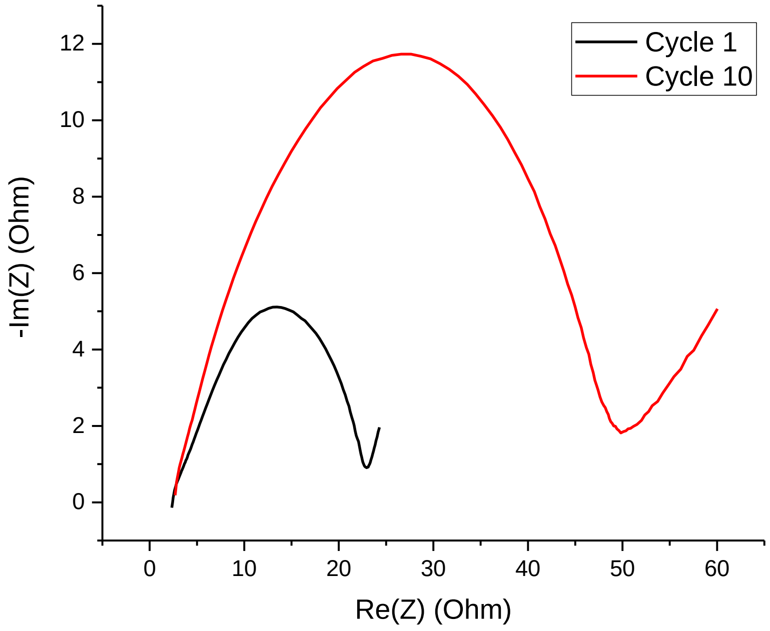

Figure 3 Impedance spectra for state-of-art graphite anode (disembled from pouch cells purchase from Lifun, China)

SPEIS study voltage profile

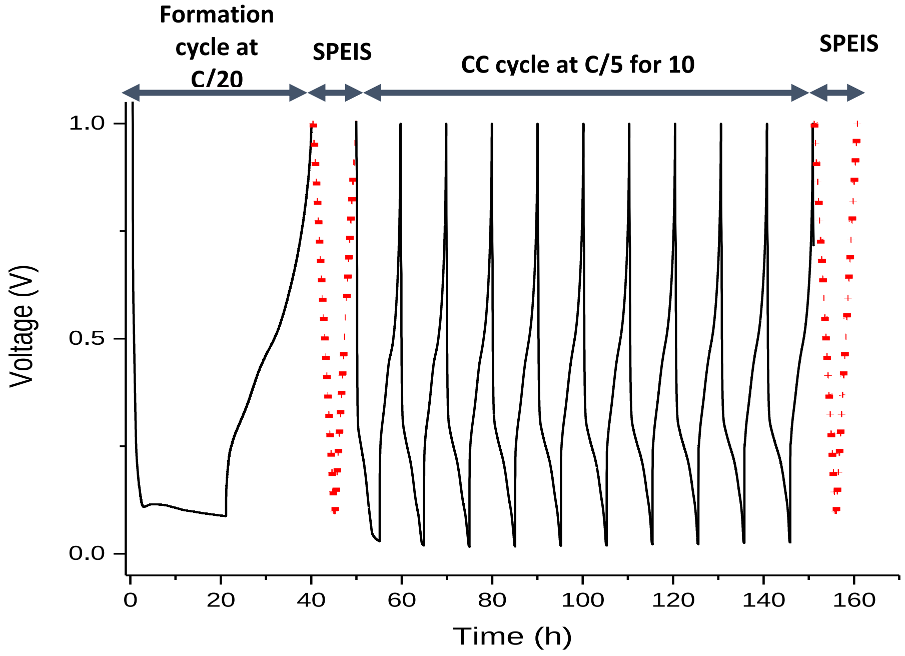

Figure 4 Continuous voltage profile for SPEIS test

Reference

1. Key, B., Morcrette, M., Tarascon, J.-M. & Grey, C. P. Pair distribution function analysis and solid state NMR studies of silicon electrodes for lithium ion batteries: understanding the (de)lithiation mechanisms. *J. Am. Chem. Soc.* **133,** 503–12 (2011).

2. Ogata, K. *et al.* Revealing lithium-silicide phase transformations in nano-structured silicon-based lithium ion batteries via in situ NMR spectroscopy. *Nat. Commun.* **5,** 3217 (2014).

3. Loveridge, M. J. *et al.* Enhancing cycling durability of Li-ion batteries with hierarchical structured silicon–graphene hybrid anodes. *Phys. Chem. Chem. Phys. Phys. Chem. Chem. Phys* **18,** 30677–30685 (2016).

4. Radvanyi, E. *et al.* Study and modeling of the Solid Electrolyte Interphase behavior on nano-silicon anodes by Electrochemical Impedance Spectroscopy. *Electrochim. Acta* **137,** 751–757 (2014).

5. Elad Pollak,* Gregory Salitra, Valentina Baranchugov, and D. A. In Situ Conductivity, Impedance Spectroscopy, and Ex Situ Raman Spectra of Amorphous Silicon during the Insertion/Extraction of Lithium. *J. Phys. Chem. C* **111,** 11437–11444 (2007).

6. Guo, J., Sun, A., Chen, X., Wang, C. & Manivannan, A. Cyclability study of silicon–carbon composite anodes for lithium-ion batteries using electrochemical impedance spectroscopy. *Electrochim. Acta* **56,** 3981–3987 (2011).

7. Gaberscek, M., Moskon, J., Erjavec, B., Dominko, R. & Jamnik, J. The Importance of Interphase Contacts in Li Ion Electrodes: The Meaning of the High-Frequency Impedance Arc. *Electrochem. Solid-State Lett.* **11,** A170 (2008).
